# The Effects of Lexical Tone Awareness on Early Word Recognition, Word Reading, and Spelling From Dictation of Thai Children: A Longitudinal Study

**DOI:** 10.3389/fpsyg.2021.719552

**Published:** 2021-08-31

**Authors:** Therdpong Thongseiratch, Tuangporn Kraiwong, Rungpat Roengpitya

**Affiliations:** ^1^Department of Pediatrics, Faculty of Medicine, Prince of Songkla University, Songkhla, Thailand; ^2^The English Program, Faculty of Liberal Arts, Mahidol University, Bangkok, Thailand

**Keywords:** lexical tone awareness, reading, Thai, tonal languages, word recognition, spelling, dictation

## Abstract

In tonal languages such as Thai, lexical tone (the pitch of a syllable) affects word meaning. This study examined the effects of lexical tone awareness (LTA) on early word recognition and the relationship between these abilities and word reading and spelling in subsequent grades. A longitudinal design was used to assess reading-related skills in 259 Thai children, first in kindergarten (130 girls, *M*age=67.25months) and later in Grade 3 (*M*age=102.25months). In kindergarten, the children were tested on lexical tone identification and differentiation, early literacy skills, non-verbal IQ, and early word recognition. In Grade 3, they were tested on word reading and spelling from dictation. The hierarchical regression analyses showed that the lexical tone identification skills in kindergarten accounted for 2% of the unique variance in early word recognition. However, none of the LTA skills could predict word reading and spelling from dictation after controlling for other literacy-related skills. These findings suggest that LTA skill positively associated with early word recognition at the kindergarten level, but not for word reading and spelling from dictation at a Grade 3 level.

## Introduction

Longitudinal studies in which children’s literacy-related skills are investigated from kindergarten through to higher grades can provide valuable insights into children’s reading ability and help predict children who will develop reading disabilities or dyslexia (van Viersen et al., 2018; Verwimp et al., 2020). Studies have found a variety of cognitive skills in kindergarten children that predict their reading ability when they reach primary school (Torppa et al., 2006, 2010; De la Calle et al., 2020). In alphabetic languages, the predictors for individual differences in reading are phonological awareness (PA), letter knowledge, and rapid automatized naming (RAN), regardless of the language variations (Snowling et al., 2003; Caravolas et al., 2012).

Although it is clear that phonological awareness alone is not sufficient to account for children’s reading ability (Pennington, 2006; McGrath et al., 2020), most studies of alphabetic languages have found that children’s phonological awareness is the primary precursor of reading (Hulme and Snowling, 2013; Snowling and Melby-Lervåg, 2016; McGrath et al., 2020). Given this principle assumption, the predominant view for many years was that children’s reading ability at age 7years and older could be explained by observing their perception of phonetic segments, such as consonants and vowels in high frequency words at the kindergarten level (i.e., at the age of 4–6years; Lyytinen et al., 2015; Snowling and Hulme, 2020). Many earlier studies have focused on children’s perception and manipulation of the onset (the initial consonant or consonant cluster of the word) and rimes (the vowel and any consonants after the vowel), which are considered as major segments of oral languages (Bergen et al., 2012; Lin et al., 2018; McGrath et al., 2020). Surprisingly, only a few studies have explored children’s perceptions of suprasegmental features of speech, speech features that accompany or are added over consonants and vowels, such as stress, intonation, tones, or rhythmic timing (Ma et al., 2017).

Some studies have reported that stress sensitivity of children aged 5–12years also contributed to reading development in English (Calet et al., 2015; Arciuli, 2017; Lin et al., 2018). Other studies reported that, in Spain, children’s stress sensitivity (at age 6–8years) predicted non-word and text reading (Gutiérrez-Palma and Palma Reyes, 2007; Gutiérrrez-Palma et al., 2009), and, in the Netherlands, 3-year-old children at risk of reading disability showed more difficulty in perceiving and imitating stress than typically-developing children, indicating the delay in word stress acquisition in the at-risk group (De Bree et al., 2006). It can be assumed that suprasegmental features of a language can also affect children’s reading development.

In tonal languages, lexical tones are used to differentiate the meaning of monosyllabic words. Lexical tone shares the same linguistic function as lexical stress (Morén and Zsiga, 2006; Wong and Perrachione, 2007; Chung et al., 2017). For example, in the Thai language, there are five lexical tones with three level tones, namely mid (tone 1), low (tone 2), and high (tone 4), and two contour tones, rising-falling (tone 3), and falling-rising (tone 5; Abramson, 1962; Roengpitya, 2007). These lexical tones are important for children in learning how to use segments to form syllables with their particular assigned tones and how to use one or more syllables to build lexical words. Specifically, words with the same consonants and vowels carry different meanings if different lexical tones are superimposed on them. For example, in the Thai words, นา/naa/ ‘rice field’, หน่า/nàa/ ‘custard apple’, หน้า/nâa/ ‘face’, น้า/náa/ ‘aunt’, and หนา/nǎa/ ‘thick,’ all five words are monosyllabic minimal pairs with the same consonant and vowel sounds, except for the five contrastive tones superimposed on them. In other words, these five tones make all five words have different meanings.

When considering the differences in segmental and suprasegmental features of speech, it is interesting to explore the effects of lexical tone awareness (LTA) along with segmental phonological awareness on Thai children’s early word recognition, word reading, and spelling from dictation. Some studies of Chinese children explored the relationship between LTA and word recognition. However, all were cross-sectional studies or studies of short-term longitudinal relationships. For example, McBride-Chang et al. (2008) found that tone sensitivity assessed in children at the age of 4 was a good predictor of Cantonese children’s character recognition at the age of 5. Another study of 8-year-old Chinese children, found that difficulties in perceiving lexical tones were a good predictor for Chinese word reading difficulties (Cheung et al., 2009). Cheung’s study did not include any other factors that might affect Chinese word reading difficulties. Thus it could not be concluded that LTA plays an important role in the children’s reading progress, given that other literacy-related skills were not controlled.

This study aimed to explore how Thai children’s LTA, related to other literacy-related skills, affects their early word recognition, word reading, and single word spelling from dictation. The study involved Thai children from kindergarten to Grade 3 level. To the best of our knowledge, our study is the first to investigate longitudinal predictors of Thai word reading, and at the same time, the first to examine longitudinal predictors of reading skills in an alphasyllabary – a language that simultaneously represents sounds at the level of a syllable and a phoneme. Alphasyllabic languages are used in South and South East Asia, Ethiopia, Northern Africa, and some northern regions of North America (Nag and Snowling, 2012).

## Materials and Methods

### Participants

Participants were drawn from 10 primary schools with kindergartens in Hatyai, Songkhla, a typical urban city in Thailand, selected by non-probability sampling method. The research was conducted under the approval of the Institutional Review Board (IRB) of Faculty of Medicine, Prince of Songkla University, Thailand. The parents of all participants signed consent forms prior to their children’s participation.

Three hundred and thirty children (169 females) participated in this study, being tested first at the kindergarten level (*M*_age_=66.84months, *SD*=3.52) and then again when they were in the third grade (Grade 3; *M*_age_=102.25months, *SD*=3.28). All children were native speakers of the Thai language, and none had a history of developmental delay in language/speech, intellectual disability, and/or sensory impairment. At the time of the Grade 3 testing 259 children, (129 males and 130 females, *M*_age_=67.25months, *SD*=3.50) were still available for the second round of testing. Seventy-one children (21.52%) did not complete the study because they had moved to other schools and could not be contacted.

### Measurements

The measurements taken in this study were non-verbal IQ, vocabulary, letter knowledge, RAN, phonological awareness, LTA, word recognition, word reading, and single word spelling dictation.

### Non-verbal IQ

In this study, Colored Progressive Matrices (CPM) of Test of Raven et al. (1998) was used to provide a standardized non-verbal measure of general intelligence. The CPM has Thai norms for typically developing children aged 5–11years (Phattharayuttawat, 2003). It consists of three sets of 12 colored multiple-choice items that gradually increase in difficulty. Each item consists of an incomplete matrix. Children are asked to select one of six options that best complete the missing part of the matrix. Each participant’s score shows the total number of correct answers. Raw scores are converted to percentile scores to rank non-verbal intelligence based on chronological age. The CPM has been shown to have good test-retest reliability at *r*=0.80 (Raven et al., 1998) and high internal consistency (*r*=0.91; Phattharayuttawat, 2003).

### Vocabulary

The Rama Pre-Read (RPR) software program was used to assess the children’s expressive language skills at the kindergarten level (Yampratoom et al., 2017). In this program, participants are asked to name as many animals and fruits they know. The number of animals and fruit named by a participant in 1min was counted. Each participant’s score shows the total number of animals and fruit. The test-retest reliability coefficient in our sample was 0.78.

### Letter Knowledge

The letter-naming task in the RPR program was used to assess the participants’ letter knowledge based on how many of the 44 Thai letters they could name. In the Thai version of this program used in our study, the children were presented with one letter at a time on a single page and then asked to name the letter. If a child named a letter correctly, s/he gained one point. The letters were arranged by familiarity of the letters, starting with the first and most familiar letters (Yampratoom et al., 2017). The order of these letters is fixed in the RPR software, and the maximum score is 44. The Cronbach’s alpha reliability coefficient in our sample was 0.94.

### Rapid Automatized Naming

In this test, a child is presented with a laptop screen containing five rows of familiar letters, with each row containing the same five letters arranged in different orders. Before the timed test, the children were asked to name all five letters in a practice trial to ensure they could name the letters correctly. The children were then asked to identify and read aloud the 25 letters in the specified order as quickly as possible (Yampratoom et al., 2017). A child’s score was the total number of the letters divided by the total time used (x/25). This task’s test-retest reliability was 0.91.

### Phonological Awareness

The initial phoneme matching task in the RPR program was used to assess phonological awareness. Children were given three practice trials and another 10 test items in this task. For each item, a child was presented with four simple pictures. Among the four, one was the stimulus, another was the target, and the others were options. After all the pictures were named, the child was asked to identify which item from the given choices matched the same initial phoneme as the one in the stimulus. All pictures depicted monosyllabic and meaningful nouns familiar to Thai preschool children (Yampratoom et al., 2017). A participant’s score was the total number of correct answers. The Cronbach’s alpha reliability coefficient in our sample was 0.96.

### Lexical Tone Awareness

Two new Thai lexical tone awareness (LTA) tasks were developed for this study, a lexical tone identification task and a lexical tone differentiation (LTD) task. Each task had 30 test items with three practical trial items. In the lexical tone identification task, children were asked to carefully listen to each syllable and identify which pictures would match the target syllable. For example, the target word/mɔ̌ ɔ/ ‘doctor’ were read aloud in Thai to the children. Then two pictures with two separate words in a minimal pair, one picture with a doctor:/mɔ̌ ɔ/ and the other with a pot/mɔ̂ɔ/, were shown, and the children were asked to select one of the two choices. In this case, the picture of the doctor was the correct answer. In the LTD task, there were 33 sets of three syllables with the same onset and rimes but with phonological tonal contrasts. This task required the children to differentiate the phonological contrastive tones in Thai. A participant’s score was the total number of correct answers. The Cronbach’s alpha reliability coefficient for the lexical tone identification task and LTD task were 0.72 and 0.89, respectively.

### Word Recognition

This task was adapted from the Thai diagnostic test of reading disability developed by Mitranun et al. (2013), based on the pattern of the standard international test of reading, the Woodcock Reading Mastery Test-Revised (Mitranun et al., 2013). Two subtasks, visual-auditory learning and word identification, were combined to assess each child’s ability to accurately recognize Thai letters and words in print. The first task consisted of five single Thai characters. The second task consisted of six two-character Thai words. All the words were arranged in an ascending order of difficulty. The internal consistency reliability for this task was Cronbach’s *α*=0.95 (Mitranun et al., 2013).

### Word Reading

The children’s word reading ability was tested using the reading accuracy subtest of the Thai version of the Wide Range Achievement Test (WRAT-Thai). This task consists of 60 words, and participants are asked to read each word out loud. All the words were arranged in ascending order of difficulty, with testing halted following 15 consecutive errors (Sayawaranon, 1997). The internal consistency reliability for this task was Cronbach’s *α*=0.92.

### Single Word Spelling From Dictation

Single word spelling from dictation was assessed with the spelling accuracy subtest of the WRAT-Thai. This task consisted of 50 words. The participants were required to write each word as dictated into the given blank space (Sayawaranon, 1997). A participant’s score was the total number of correct words. The internal consistency reliability for this task was Cronbach’s *α*=0.85.

### Procedure

All participants were tested individually in a separate room in their school during school hours by a child psychologist who had experiences in psychological testing. The first testing phase (from June to July 2016) was carried out when the children were in kindergarten. All children were assessed on six literacy/cognitive tasks at this time. About 3years later (July to October 2019), they were contacted again for assessment of word reading and spelling from dictation. At the kindergarten level, the tests lasted approximately 45min. At the Grade 3 level, the tests lasted 1h. Following the IRB guidelines, the informed consent was obtained from all children’s parents before each testing phase. For the statistical analyses, SPSS 26.0 was used to conduct descriptive and regression analyses.

## Results

The results are presented in two sections: descriptive analysis and prediction of word recognition, reading, and spelling from dictation.

### Descriptive Analysis

The means and SDs for all measures used in our study are shown in Table 1. There were no missing data, and all the analyses were performed with a full dataset of 259 participants. An examination of the measures’ distributional properties revealed that they were within acceptable levels.

**Table 1:** Correlations between the variables.

S.no	Measures	1	2	3	4	5	6	7	8	9	10	11	
**Kindergarten**												
1.	Non-verbal IQ											
2.	Vocabulary	0.25										
3.	Education	0.26	0.31									
4.	Letter knowledge	0.34	0.36	0.32								
5.	RAN	0.30	0.36	0.40	0.61							
6.	PA	0.27	0.28	0.35	0.32	0.42						
7.	LTI	0.33	0.29	0.33	0.34	0.40	0.35					
8.	LTD	0.39	0.27	0.34	0.36	0.40	0.36	0.42				
9.	Word recognition	0.29	0.37	0.41	0.56	0.54	0.45	0.43	0.39			
	**Grade 3**												
10.	Word reading	0.38	0.38	0.38	0.52	0.53	0.40	0.36	0.34	0.54		
11.	Word spelling from dictation	0.38	0.40	0.36	0.57	0.51	0.38	0.37	0.33	0.55	0.87	
	Maximum scores	36	44	–	44	0.83	10	30	30	13	57	44
	*Mean*	27.19	17.64	–	36.32	0.41	5.19	20.83	17.73	7.37	33.81	22.70
	*SD*	5.71	5.77	–	7.91	0.13	2.30	3.62	6.90	2.73	12.98	9.72

RAN, rapid automatized naming; PA, phonological awareness; LTI, lexical tone identification; LTD, lexical tone differentiation; for all correlation *r*’s, *p*<0.001.

Table 1 shows the correlations among all variables used in the following hierarchical regression analyses. Both measures of LTA correlated moderately with each other (*r*=0.42). Both tests also correlated moderately with word recognition, word reading, and word spelling from dictation at the Grade 3 level (*r*s ranged from 0.33 to 0.43). However, the correlation with word recognition at the kindergarten level (*r*s ranged from 0.39 to 0.43) was generally higher than the correlation with word reading and word spelling from dictation at the Grade 3 level (*r*s ranged from 0.33 to 0.37).

### Predicting Word Recognition, Reading, and Spelling From Dictation

We further investigated how children’s LTA skills would affect early word recognition and their future word reading and spelling from dictation by carrying out three sets of hierarchical regression analyses separately for word recognition at the kindergarten level, and word reading and spelling from dictation at the Grade 3 level (Table 2).

**Table 2:** Hierarchical regression analyses with measures in the Kindergarten level as predictors of word recognition.

S.no	Measures	Model 1		Model 2		Model 3	
		*β*	95%CI	*β*	95%CI	*β*	95%CI
1.	Non-verbal IQ	0.15^**^	0.01–0.05	0.02	−0.02–0.02	−0.01	−0.02–0.02
2.	Vocabulary	0.25^***^	0.06–0.17	0.10	−0.01–0.09	0.08	−0.01–0.09
3.	Mother’s education	0.30^***^	0.62–1.40	0.14^**^	0.01–0.12	0.12^*^	0.05–0.77
4.	Letter sound knowledge			0.30^***^	0.06–0.15	0.29^***^	0.06–0.14
5.	RAN			0.18^**^	1.00–6.20	0.14^*^	0.32–5.57
6.	Phonological awareness			0.20^***^	0.12–0.37	0.18^**^	0.08–0.34
7.	Lexical tone identification					0.14^*^	0.02–0.19
8.	Lexical tone differentiation					0.05	−0.03–0.06
	*R* Square	0.26^***^		0.46^***^		0.48^***^	

* *p*<0.05;

** *p*<0.01;

*** *p*<0.001.

In all regression analyses, non-verbal IQ, vocabulary, and mother’s education were entered as control variables in Model 1 of the regression equation. In Model 2, a set of literacy-related skills, letter-sound knowledge, RAN, and phonological awareness, were entered. In Model 3, the LTA skills were entered as a set to evaluate the shared and unique contributions. Tables 2–4 report the *R*^2^, standardized betas, and significance levels of each step of the regression analysis for word recognition, reading, and spelling from dictation, respectively.

#### Word Recognition in Kindergarten

The first set of analyses examined how children’s LTA skills affected word recognition in kindergarten relative to other literacy-related skills. The LTA skills accounted for a significant amount of variance in word recognition (Table 2). The joint contribution of the two LTA skills to word recognition did not substantially surpass their individual contribution. However, only lexical tone identification was a significant predictor of word recognition when entered as a set of LTA skills and at the same step with the rest of the LTA skills. The literacy-related skills accounted for a significant 20% of variance in word recognition, after the effects of non-verbal IQ, vocabulary, and mother’s education were controlled. The standardized beta coefficients further confirmed that letter-sound knowledge (*β*=0.30), phonological awareness (*β*=0.20), RAN (*β*=0.18), and lexical tone identification (*β*=0.14) were the significant predictors associated with children’s early word recognition.

#### Word Reading in Grade 3

The second set of analyses was performed to evaluate how children’s LTA could affect word reading in Grade 3, as opposed to other literacy-related skills. The results showed that none of the LTA skills significantly predicted word reading in Grade 3 (Table 3). In contrast, the literacy-related skills accounted for a significant 14% of variance in word reading, after the effects of non-verbal IQ, vocabulary, and mother’s education were controlled. The standardized beta coefficients further confirmed that letter-sound knowledge (*β*=0.22), RAN (*β*=0.21), and phonological awareness (*β*=0.13) were the three significant predictors associated with children’s word reading in Grade 3.

**Table 3:** Hierarchical regression analyses with measures in the Kindergarten level as predictors of word reading in Grade 3.

S.no	Measures	Model 1		Model 2		Model 3	
		*β*	95%CI	*β*	95%CI	*β*	95%CI
1.	Non-verbal IQ	0.26^***^	0.13–0.34	0.15^**^	0.04–0.23	0.14^*^	0.03–0.23
2.	Vocabulary	0.25^***^	0.30–0.81	0.12^*^	0.03–0.52	0.12^*^	0.02–0.51
3.	Mother’s education	0.23^***^	1.86–5.52	0.10	−0.18–3.34	0.10	−0.28–3.30
4.	Letter sound knowledge			0.22^**^	0.15–0.56	0.22^**^	0.15–0.56
5.	RAN			0.21^**^	7.55–32.90	0.20^**^	6.57–32.47
6.	Phonological awareness			0.13^*^	0.13–1.37	0.13^*^	0.08–1.35
7.	Lexical tone identification					0.04	−0.25–0.57
8.	Lexical tone differentiation					−0.01	−0.23–0.21
	*R* Square	0.28^***^		0.42^***^		0.42^***^	

* *p*<0.05;

** *p*<0.01;

*** *p*<0.001.

#### Word Spelling From Dictation in Grade 3

The final set of analyses was performed to evaluate how children’s LTA could affect word spelling from dictation in Grade 3, in contrast with other literacy-related skills. Again, the results showed that none of the LTA skills was a significant predictor of word spelling from dictation in Grade 3 (Table 4). The literacy-related skills accounted for a significant 15% of variance in word spelling from dictation, after the effects of non-verbal IQ, vocabulary, and mother’s education were controlled. However, the standardized beta coefficients showed that letter-sound knowledge (*β*=0.32) and RAN (*β*=0.15) were the two significant predictors associated with children’s word spelling from dictation in Grade 3, while phonological awareness was not.

**Table 4:** Hierarchical regression analyses with measures in the Kindergarten level as predictors of word spelling from dictation in Grade 3.

S.no	Measures	Model 1		Model 2		Model 3		*β*	95%CI	*β*	95%CI	*β*	95%CI
1.	Non-verbal IQ	0.26^***^	0.10–0.25	0.14^*^	0.03–0.17	0.13^*^	0.02–0.17
2.	Vocabulary	0.28^***^	0.27–0.65	0.14^*^	0.06–0.42	0.14^*^	0.05–0.41
3.	Mother’s education	0.21^***^	1.10–3.85	0.08	−0.32–2.30	0.08	−0.40–2.25
4.	Letter sound knowledge			0.32^***^	0.23–0.54	0.31^***^	0.23–0.54
5.	RAN			0.15^*^	1.36–20.16	0.14^*^	0.46–19.62
6.	Phonological awareness			0.10	−0.03–0.89	0.10	−0.07–0.87
7.	Lexical tone identification					0.08	−0.09–0.52
8.	Lexical tone differentiation					−0.03	−0.20–0.12
	*R* Square	0.28^***^		0.43^***^		0.43^***^	

* *p*<0.05;

*** *p*<0.001.

## Discussion

This study examined the effects of the Thai LTA on early word recognition, word reading, and word spelling from dictation. The roles of other key existing predictors (letter knowledge, RAN, and phonological awareness) all together were also investigated. Although the results of this study indicated that LTA had a significant effect on early word recognition, which was estimated together with other predictors while the child was in kindergarten, it was not a predictor for word reading and single word spelling from dictation in Grade 3 children.

The results of this study concur with Tong et al. (2015). Their cross-sectional study examined the role of LTA and other literacy-related skills in children aged 5–6years. These children had Cantonese as their first language, and it was found that LTA was related to word recognition after other related factors were controlled. The current study was also consistent with an earlier study of English-speaking adults using Mandarin or Cantonese as a second language that also found that LTA was a significant predictor of word recognition (Wong and Perrachione, 2007; Tong et al., 2015).

However, our study advanced the findings of Tong et al. (2015) by using more tests to study both types of LTA: (1) the lexical tone identification task drawing on high-level lexical pitch abilities, i.e., the tone-semantic association of known words, and (2) the LTD task, which assessed low-level or non-lexical pitch abilities unrelated to word meaning, i.e., a pure tone perception task at the acoustic level. Previous work with adults examined only pure tone perception, which was found to play an important role in word recognition prediction (Cooper and Wang, 2012). The study of preschool children of Tong et al. (2015) specifically examined lexical tone identification tasks, which were also found to play a role in recognition prediction.

The current findings extend previous work that found that, at the kindergarten level, lexical tone identification only plays an important role in word recognition. In contrast, pure tone perception did not have a significant role after other key factors were controlled. These results highlight that learning to read is closely related to learning vocabulary. Our findings confirmed the “pitch to semantic mapping” hypothesis for reading tonal languages (Nguyễn et al., 2008). The results also affirmed the relationship between suprasegmental phonology and word reading according to recent word reading theory, which proposed a link between suprasegmental phonology and word reading (Arciuli et al., 2010). Our findings are consistent with studies that have indicated that English stress sensitivity was similarly associated with word recognition (Holliman et al., 2010; Goswami et al., 2013). Our study complemented this knowledge in an alphasyllabary language where lexical tones were also used to differentiate word meanings.

When considering other literacy-related skills of the Thai kindergarteners in our study, the skills that significantly affected word recognition were letter knowledge, phonological awareness, RAN, and LTA. However, when considering the longitudinal findings at the Grade 3 level, LTA did not play a role in word reading or word spelling from dictation. However, there were still three predictive factors, namely, letter knowledge (the most important predictor), followed by RAN, and phonological awareness.

It could be concluded that both segmental and suprasegmental phonological awareness had limited predictive abilities for both reading and spelling when the children had advanced to primary level. These general findings corresponded to a longitudinal study in native Cantonese children, which also found that LTA did not affect word reading prediction when children had advanced to primary level (McBride-Chang et al., 2011).

Given the unique educational challenges in a country using an alphasyllabary (Vibulpatanavong and Evans, 2019), formal literacy instruction usually begins at a very early age, and children learn the alphabet earlier than usual. This makes letter knowledge and RAN better predictors for word reading and word spelling from dictation at the higher (primary school) level when compared to both segmental and suprasegmental phonological awareness. Children learn letters and recognize letter shapes before they learn the phonological sounds. In Thailand and other countries where the native language is an alphasyllabary, children learn how to read by learning the alphabet first. Most schools in Thailand do not provide any type of phonic-based instruction. When children learn letters in the early grades, they learn how to combine words with consonants and vowels and learn Thai tones without systematic phonics instruction. Thus, neither segmental nor suprasegmental phonological awareness measured at the kindergarten level is a useful predictor for later word reading and word spelling from dictation.

In terms of clinical implications, it can be concluded that neither LTA test is particularly useful as a predictor of later word reading or word spelling from dictation. We did find a significant role of both letter knowledge and RAN as predictors for word reading and word spelling from dictation in Grade 3, which might then also have some use in predicting the possibility of a child having or developing a reading disability.

There are some limitations to this study. First, because of the limited reading tests available in Thailand, literacy skills can be measured only through word reading and word spelling from dictation. However, another test is planned for future assessment of reading fluency and comprehension. Second, this study assessed phonological awareness using only initial-sound matching, which may not represent all phonological awareness skills. Third, this study evaluated RAN only for letter naming, which may not have been the most appropriate choice as not all children can say their letters clearly at the kindergarten level. However, as noted, formal literacy instruction begins at a very early age in Thailand; thus, we felt this task would be useful. Fourth, although our study has evidenced that LTA played a role in word recognition and that their relations may vary according to children’s characteristics, family background, or even school-level factors. Moreover, various relationships among all direct and indirect variables (e.g., LTA, PA, and vocabulary) that could be either direct or indirect can be shown in a path diagram. Structural equation modeling should be studied to provide an extension to multiple regressions of our finding, particularly when involving more variables to produce path analysis.

Despite these limitations, this study was the first longitudinal study to examine the reading predictors in an alphasyllabary language and also longitudinal predictors which assessed lexical tones, both the lexical tone identification task with a focus on the lexical tone identification of familiar words and the pure tone perception task, as predictors of future reading skills.

## Conclusion

Our study contributes to a growing body of research on different typological groups of languages showing that LTA skills are important in the early phase of reading development in children but not for reading development at the primary level. Our findings showed the joint contribution of letter knowledge and RAN to word reading and spelling from dictation. This suggests further studies are required to assess the degree of letter knowledge and RAN at the kindergarten level, ultimately enabling more effective early identification of children at risk of reading disability.

## Data Availability Statement

The original contributions presented in the study are included in the article/supplementary material, further inquiries can be directed to the corresponding author.

## Ethics Statement

The research was conducted under the approval of the Institutional Review Board (IRB) of Faculty of Medicine, Prince of Songkla University, Thailand. The parents of all participants signed consent forms prior to their children’s participation.

## Author Contributions

TT designed and performed experiments, analyzed data, and co-wrote the paper. TK performed experiments. RR analyzed data and co-wrote the paper. All authors contributed to the article and approved the submitted version.

## Conflict of Interest

The authors declare that the research was conducted in the absence of any commercial or financial relationships that could be construed as a potential conflict of interest.

## Publisher’s Note

All claims expressed in this article are solely those of the authors and do not necessarily represent those of their affiliated organizations, or those of the publisher, the editors and the reviewers. Any product that may be evaluated in this article, or claim that may be made by its manufacturer, is not guaranteed or endorsed by the publisher.

## References

[ref1] Abramson A. (1962). The vowels and tones of standard thai: acoustical measurements and experiments. J. Int. Am. Linguist. 28, 1–143.

[ref2] Arciuli J. (2017). The relationship between children’s sensitivity to dominant and non-dominant patterns of lexical stress and reading accuracy. J. Exp. Child Psychol. 157, 1–13. 10.1016/j.jecp.2016.11.016, PMID: 28088677

[ref3] Arciuli J. Monaghan P. Seva N. (2010). Learning to assign lexical stress during reading aloud: corpus, behavioral, and computational investigations. J. Mem. Lang. 63, 180–196. 10.1016/j.jml.2010.03.005

[ref4] Bergen E. Jong P. F. Plakas A. Maassen B. A. M. Leij A. (2012). Child and parental literacy levels within families with a history of dyslexia. J. Child Psychol. Psychiatry 53, 28–36. 10.1111/j.1469-7610.2011.02418.x, PMID: 21615405

[ref5] Calet N. Gutiérrez-Palma N. Simpson I. C. González-Trujillo M. C. Defior S. (2015). Suprasegmental phonology development and reading acquisition: a longitudinal study. Sci. Stud. Read. 19, 51–71. 10.1080/10888438.2014.976342

[ref6] Caravolas M. Lervåg A. Mousikou P. Efrim C. Litavský M. Onochie-Quintanilla E.. (2012). Common patterns of prediction of literacy development in different alphabetic orthographies. Psychol. Sci. 23, 678–686. 10.1177/0956797611434536, PMID: 22555967 PMC3724272

[ref7] Cheung H. Chung K. K. H. Wong S. W. L. McBride-Chang C. Penney T. B. Ho C. S. H. (2009). Perception of tone and aspiration contrasts in chinese children with dyslexia. J. Child Psychol. Psychiatry 50, 726–733. 10.1111/j.1469-7610.2008.02001.x, PMID: 19175808

[ref8] Chung W. Jarmulowicz L. Bidelman G. M. (2017). Auditory processing, linguistic prosody awareness, and word reading in mandarin-speaking children learning english. Read. Writ. 30, 1407–1429. 10.1007/s11145-017-9730-8

[ref9] Cooper A. Wang Y. (2012). The influence of linguistic and musical experience on cantonese word learning. J. Acoust. Soc. Am. 131, 4756–4769. 10.1121/1.4714355, PMID: 22712948

[ref10] De Bree E. Wijnen F. Zonneveld W. (2006). Word stress production in three-year-old children at risk of dyslexia. J. Res. Read. 29, 304–317. 10.1111/j.1467-9817.2006.00310.x

[ref11] De la Calle A. M. Guzmán-Simón F. García-Jiménez E. Aguilar M. (2020). Precursors of reading performance and double- and triple-deficit risks in spanish. J. Learn. Disabil. 54:22219420979960. 10.1177/0022219420979960 33355031

[ref12] Goswami U. Mead N. Fosker T. Huss M. Barnes L. Leong V. (2013). Impaired perception of syllable stress in children with dyslexia: a longitudinal study. J. Mem. Lang. 69, 1–17. 10.1016/j.jml.2013.03.001

[ref13] Gutiérrez-Palma N. Palma Reyes A. (2007). Stress sensitivity and reading performance in spanish: a study with children. J. Res. Read. 30, 157–168. 10.1111/j.1467-9817.2007.00339.x

[ref14] Gutiérrrez-Palma N. Raya-García M. Palma-Reyes A. (2009). Detecting stress patterns is related to children's performance on reading tasks. Appl. Psycholinguist. 30, 1–21. 10.1017/S0142716408090012

[ref15] Holliman A. J. Wood C. Sheehy K. (2010). Does speech rhythm sensitivity predict children's reading ability 1 year later? J. Educ. Psychol. 102, 356–366. 10.1037/a0018049

[ref16] Hulme C. Snowling M. J. (2013). Learning to read: what we know and what we need to understand better. Child Dev. Perspect. 7, 1–5. 10.1111/cdep.12005, PMID: 26290678 PMC4538787

[ref17] Lin C. Y. Wang M. Newman R. S. Li C. (2018). The development of stress sensitivity and its contribution to word reading in school-aged children. J. Res. Read. 41, 259–277. 10.1111/1467-9817.12094, PMID: 30270946 PMC6162047

[ref18] Lyytinen H. Erskine J. Hämäläinen J. Torppa M. Ronimus M. (2015). Dyslexia—early identification and prevention: highlights from the jyväskylä longitudinal study of dyslexia. Curr. Dev. Disord. Rep. 2, 330–338. 10.1007/s40474-015-0067-1, PMID: 26543798 PMC4624816

[ref19] Ma W. Zhou P. Singh L. Gao L. (2017). Spoken word recognition in young tone language learners: age-dependent effects of segmental and suprasegmental variation. Cognition 159, 139–155. 10.1016/j.cognition.2016.11.011, PMID: 27951429

[ref20] McBride-Chang C. Lam F. Lam C. Chan B. Fong C. Y. Wong T. T.. (2011). Early predictors of dyslexia in Chinese children: familial history of dyslexia, language delay, and cognitive profiles: early predictors of dyslexia in Chinese children. J. Child Psychol. Psychiatry 52, 204–211. 10.1111/j.1469-7610.2010.02299.x, PMID: 20735514

[ref42] McBride-Chang C. Lam F. Lam C. Doo S. Wong S. W. Chow Y. Y. (2008). Word recognition and cognitive profiles of Chinese pre-school children at risk for dyslexia through language delay or familial history of dyslexia. J. Child. Psychol. Psychiatry 49, 211–218. 10.1111/j.1469-7610.2007.01837.x 17979961

[ref21] McGrath L. M. Peterson R. L. Pennington B. F. (2020). The multiple deficit model: progress, problems, and prospects. Sci. Stud. Read. 24, 7–13. 10.1080/10888438.2019.1706180, PMID: 32440085 PMC7241589

[ref22] Mitranun C. Kajornsin B. Arrayavinyoo P. (2013). The development of diagnostic reading test for children with reading disabilities in grade 3. J. Educ. Res. 8, 37–54.

[ref23] Morén B. Zsiga E. (2006). The lexical and post-lexical phonology of thai tones. Nat. Lang. Linguist. Theory 24, 113–178. 10.1007/s11049-004-5454-y

[ref24] Nag S. Snowling M. J. (2012). Reading in an alphasyllabary: implications for a language universal theory of learning to read. Sci. Stud. Read. 16, 404–423. 10.1080/10888438.2011.576352

[ref25] Nguyễn T. A. Ingram C. L. J. Pensalfini J. R. (2008). Prosodic transfer in vietnamese acquisition of english contrastive stress patterns. J. Phon. 36, 158–190. 10.1016/j.wocn.2007.09.001

[ref26] Pennington B. F. (2006). From single to multiple deficit models of developmental disorders. Cognition 101, 385–413. 10.1016/j.cognition.2006.04.008, PMID: 16844106

[ref27] Phattharayuttawat S. (2003). The normative score of the colored progressive matrices for the thai population. J. Clin. Psychol. 34, 58–70.

[ref28] Raven J. C. Court J. H. Raven J. E. (1998). Manual for Raven's Progressive Matrices and Vocabulary Scales. Bloomington, MN: Pearson

[ref29] Roengpitya R. (2007). “The variations, quantification, and generalizations of standard Thai tones,” in Experiment Approaches to Phonology. eds. Solé M.-J., Beddor P. Ohala M. (New York: Oxford University Press), 270–301.

[ref30] Sayawaranon P. (1997). A study of academic achievement in primary school students. J. Clin. Psychol. 28, 24–37.

[ref31] Snowling M. J. Gallagher A. Frith U. (2003). Family risk of dyslexia is continuous: individual differences in the precursors of reading skills. Child Dev. 74, 358–373. 10.1111/1467-8624.7402003, PMID: 12705560

[ref32] Snowling M. J. Hulme C. (2020). Annual research review: reading disorders revisited—the critical importance of oral language. J. Child Psychol. Psychiatry 62, 635–653. 10.1111/jcpp.13324, PMID: 32956509

[ref33] Snowling M. J. Melby-Lervåg M. (2016). Oral language deficits in familial dyslexia: a meta-analysis and review. Psychol. Bull. 142, 498–545. 10.1037/bul0000037, PMID: 26727308 PMC4824243

[ref34] Tong X. Tong X. McBride-Chang C. (2015). Tune in to the tone: lexical tone identification is associated with vocabulary and word recognition abilities in young chinese children. Lang. Speech 58, 441–458. 10.1177/0023830914562988, PMID: 27483739

[ref35] Torppa M. Lyytinen P. Erskine J. Eklund K. Lyytinen H. (2010). Language development, literacy skills, and predictive connections to reading in finnish children with and without familial risk for dyslexia. J. Learn. Disabil. 43, 308–321. 10.1177/0022219410369096, PMID: 20479461

[ref36] Torppa M. Poikkeus A. Laakso M. Eklund K. Lyytinen H. (2006). Predicting delayed letter knowledge development and its relation to grade 1 reading achievement among children with and without familial risk for dyslexia. Dev. Psychol. 42, 1128–1142. 10.1037/0012-1649.42.6.1128, PMID: 17087547

[ref37] van Viersen S. de Bree E. H. Zee M. Maassen B. van der Leij A. de Jong P. F. (2018). Pathways into literacy: the role of early oral language abilities and family risk for dyslexia. Psychol. Sci. 29, 418–428. 10.1177/0956797617736886, PMID: 29346032 PMC5862320

[ref38] Verwimp C. Vanden B. F. Kellens S. Economou M. Vandermosten M. Wouters J.. (2020). Pre-literacy heterogeneity in dutch-speaking kindergartners: latent profile analysis. Ann. Dyslexia 70, 275–294. 10.1007/s11881-020-00207-9, PMID: 33074483

[ref39] Vibulpatanavong K. Evans D. (2019). Phonological awareness and reading in thai children. Read. Writ. 32, 467–491. 10.1007/s11145-018-9867-0

[ref40] Wong P. C. M. Perrachione T. K. (2007). Learning pitch patterns in lexical identification by native english-speaking adults. Appl. Psycholinguist. 28, 565–585. 10.1017/S0142716407070312

[ref41] Yampratoom R. Aroonyadech N. Ruangdaraganon N. Roongpraiwan R. Kositprapa J. (2017). Emergent literacy in thai preschoolers: a preliminary study. J. Dev. Behav. Pediatr. 38, 395–400. 10.1097/DBP.0000000000000457, PMID: 28661956

